# Solubilization and Stabilization of Isolated Photosystem I Complex with Lipopeptide Detergents

**DOI:** 10.1371/journal.pone.0076256

**Published:** 2013-09-30

**Authors:** Xiaoqiang Wang, Guihong Huang, Daoyong Yu, Baosheng Ge, Jiqian Wang, Fengxi Xu, Fang Huang, Hai Xu, Jian R. Lu

**Affiliations:** 1 State Key Laboratory of Heavy Oil Processing and Center for Bioengineering and Biotechnology, China University of Petroleum (East China), Qingdao, Shandong, P. R. China; 2 Biological Physics Laboratory, School of Physics and Astronomy, University of Manchester, Manchester, United Kingdom; University of Hyderabad, India

## Abstract

It is difficult to maintain a target membrane protein in a soluble and functional form in aqueous solution without biological membranes. Use of surfactants can improve solubility, but it remains challenging to identify adequate surfactants that can improve solubility without damaging their native structures and biological functions. Here we report the use of a new class of lipopeptides to solubilize photosystem I (PS-I), a well known membrane protein complex. Changes in the molecular structure of these surfactants affected their amphiphilicity and the goal of this work was to exploit a delicate balance between detergency and biomimetic performance in PS-I solubilization via their binding capacity. Meanwhile, the effects of these surfactants on the thermal and structural stability and functionality of PS-I in aqueous solution were investigated by circular dichroism, fluorescence spectroscopy, SDS-PAGE analysis and O_2_ uptake measurements, respectively. Our studies showed that the solubility of PS-I depended on both the polarity and charge in the hydrophilic head of the lipopeptides and the length of its hydrophobic tail. The best performing lipopeptides in favour of PS-I solubility turned out to be C14DK and C16DK, which were comparable to the optimal amphiphilicity of the conventional chemical surfactants tested. Lipopeptides showed obvious advantages in enhancing PS-I thermostability over sugar surfactant DDM and some full peptide amphiphiles reported previously. Fluorescence spectroscopy along with SDS-PAGE analysis demonstrated that lipopeptides did not undermine the polypeptide composition and conformation of PS-I after solubilization; instead they showed better performance in improving the structural stability and integrity of this multi-subunit membrane protein than conventional detergents. Furthermore, O_2_ uptake measurements indicated that PS-I solubilized with lipopeptides maintained its functionality. The underlying mechanism for the favorable actions of lipopeptide in PS-I solubilization and stabilization is discussed.

## Introduction

Surfactants play important roles in membrane protein research, from isolation, purification, crystallization, structural determination to functional studies [1-5]. Initially, the use of surfactants is to disrupt biological membranes in which membrane proteins are embedded and maintain proteins in a solubilized form away from the complex native environment. Recently, the burgeoning structural and functional studies generate huge demands for the surfactant-solubilized membrane proteins in a functional, properly folded state.

Although the significance of surfactants for the study of membrane proteins has long been recognized, the exact way by which surfactants interact with membrane proteins still remains unclear. Our knowledge of the fundamental processes by which surfactants mediate membrane protein’s solubilization and stabilization is currently limited. As a result, labor-intensive screening is typically required to obtain an appropriate one from a multitude of surfactants/detergents for a membrane protein of interest and a specific purpose [6-8]. Despite these disadvantages, mounting experience and some general trends observed in this field can be useful as guides for surfactant selection. For example, surfactants with small and charged heads like sodium dodecyl sulfate (SDS) are highly effective in the solubilization of membrane proteins but tend to be denaturing, associated with unfolding of native protein structures; surfactants with large and nonionic heads like *n*-dodecyl-*β*-D-maltopyranoside (DDM) are mild and non-denaturing but the resulting large protein-detergent complexes disfavor the structural analyses [5,9-11].

In the past 10 years, it has also become evident that surfactants closely mimicking the biological properties of the lipid bilayers will be more suitable for maintaining the structural integrity and functionality of a target membrane protein. Inspired by the self-assembly of lipids, Zhang et al have designed the lipid-like peptides, consisting of a hydrophobic tail of three or more consecutive hydrophobic amino acid residues and a polar head of one or two hydrophilic residue(s). Some of them (e.g. ac-A _6_K-CONH_2_, ac-A _6_D-CONH_2_, and ac-I_5_K_2_-CONH_2_) have showed better effectiveness than conventional surfactants like n-octyl-β-D-glucopyranoside (OG) and DDM in the stabilization of membrane proteins such as functional Photosystem-I (PS-I) and G-protein coupled receptors (GPCRs) [12-14]. Lipopeptides are another class of surfactants with biological origin, consisting of a peptide sequence as the head group covalently attached to a fatty acid moiety [15-17]. Due to the combination of characteristics of lipids and peptides, they possess high membrane-like features. Furthermore, the great diversities in amino acids and peptide sequences favor easy variations in the hydrophilic head group of lipopetides, and the design of the head group has a marked influence on the interactions of the lipopeptides with membrane proteins. Recently, lipopeptides containing two alkyl chains separated by an amphipathic α-helical peptide have been designed and employed to stabilize integral membrane proteins containing α-helices and β-barrels [18]. In spite of the effectiveness of these lipopeptides, their structural complexity and the length of the 25 peptide residues make them very expensive to produce in large scale, thus limiting their widespread use.

In this study, we describe the design and synthesis of a series of ultrashort lipopeptides consisting of only two amino acid residues. In spite of the untrashort length of the dipeptide moiety, the pair-wise combination of three amino acids (e.g., Gly, Asp and Lys) still produces considerable variations in their head groups and thus their physicochemical properties. Their CMCs and ability to solubilize and stabilize the PS-I membrane protein isolated from the thylakoid membranes of *Spirulina* *platensis* are assessed.

Located in thylakoid membranes of cyanobacteria, algae and plants, photosystem-I (PS-I) is a supramolecular membrane protein complex catalyzing light reactions in photosynthesis [19]. This membrane protein is one of nature’s most efficient light harvesting complexes and thus offers an exciting paradigm for the development of biomimetic solar energy harvesting devices as well as hydrogen and electrical current generators [20,21]. As a result, considerable attention has been paid to PS-I in the current pursuit of renewable energy that is expected to replace fossil fuels in the foreseeable future. For instance, PS-I and cytochrome-c_6_, in combination with a platinum catalyst, have been utilized to generate hydrogen through photosynthesis, achieving a hydrogen yield much greater than biomass-to-fuel strategies [21]. It is a crucial step to select appropriate surfactants that facilitate the solubilization and stabilization of PS-I in such bioenergy and biotechnology research. Several studies have demonstrated the potential of peptide-based surfactants in addressing these issues with various target membrane proteins [12-14,22-25].

## Results

### Design of lipopeptides and their CMCs determination

**Figure 1:**
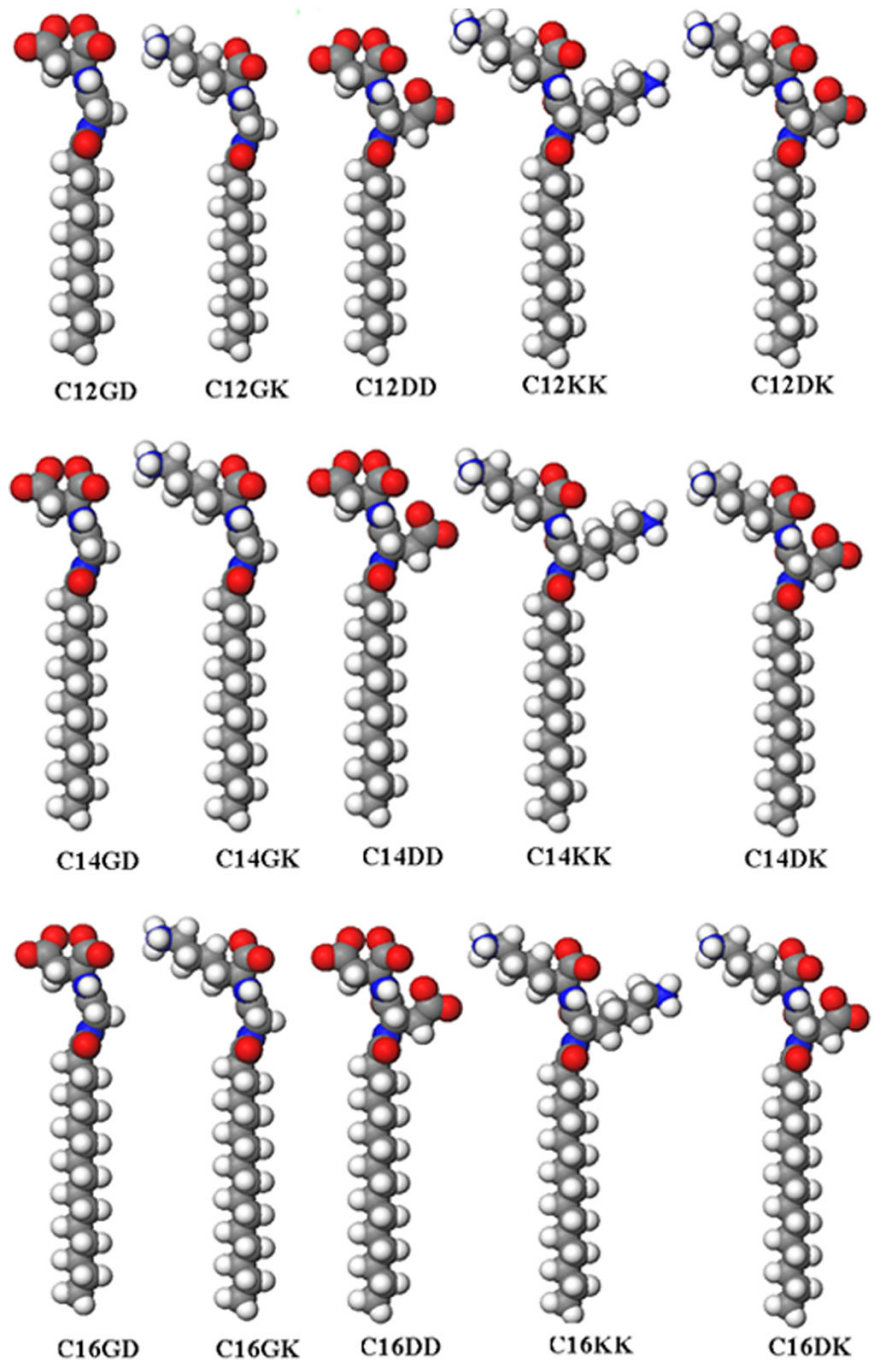
Molecular models of the designed lipopeptides. The hydrophilic heads of these surfactants vary through the pair-wise combination of hydrophilic amino acids Gly (G), Asp (D) and Lys (K), and their hydrophobic tails consist of the alkyl chains of lauric acid, myristic acid or palmitic acid, as indicated by C12, C14 and C16, respectively. Each row lists the lipopeptides with the same hydrophobic tails but different hydrophilic heads, whereas each column lists those with the same hydrophilic heads but different hydrophobic tails. Color code: gray, carbon; red, oxygen; blue, nitrogen; and white, hydrogen.

The molecular models of designed lipopeptides are shown in Figure 1. To avoid long peptide sequences and reduce the production cost, dipeptides were employed as the heads of lipopeptides with sufficient varieties and features. By the pair-wise combination of hydrophilic amino acids Gly (G), Asp (D) and Lys (K), five different hydrophilic heads GD, GK, DK, DD and KK were obtained. Although very short in length, these peptide moieties are still able to provide considerable variations in their physiochemical characteristics, in particular, charge and charge distribution and size. The N termini of the dipeptide sequences were blocked by fatty acids through the formation of an amide bond. Overall, because fatty acids can be regarded as a building block like amino acids in the peptide synthesis, the synthesis of these designed lipopetides is very simple and consists of only three coupling reaction steps. The use of fatty acids with different alkyl chain lengths results in marked variations in the critical micelles concentration (CMCs). In this study, lauric acid, myristic acid and palmitic acid were used, termed as C12, C14 and C16, respectively.

In order to solubilize membrane proteins effectively, surfactants are typically used at concentrations above their CMCs [4,5]. By measuring surface tension against concentrations of the lipopeptides, their CMCs were determined in Tris-HCl buffer and mostly fell in a broad range from 0.03 to 5.0 mM, depending on the specific molecular composition (Table 1). These values are comparable to those of many conventional detergents such as SDS, DDM, LDAO, Triton X-100, and DPC, which are often employed in the solubilization of membrane proteins. Furthermore, increase in the length of the alkyl chain of the lipopeptides by two methylene groups led to an approximately 10-fold reduction in CMC, similar to the conventional ionic detergents. In contrast, variations in the hydrophilic peptide moiety had far weaker influence on the CMCs. The lipopeptides with the same alkyl chain had close CMC values, except for the one carrying DD, whose CMC increased by about one order of magnitude. Note that C16KK was prone to precipitation after a prolonged period of time in Tris-HCl buffer.

**Table 1:** Properties of the lipopeptides and those from the two popular conventional surfactants are also listed for comparison.

Detergent	Mw (Da)_theoretical_^1^	Mw (Da)_MS_^2^	Net charge, pH 7.0	CMC (mM)
C12GD	372.5	395.1	-2	5
C12GK	386.2	386.2	0	4
C12DD	431.1	431.1	-3	17
C12KK	457.3	457.3	1	2
C12DK	443.6	426.2	-1	5
C14GD	400.5	423.2	-2	0.35
C14GK	414.5	414.3	0	0.5
C14DD	458.5	481.2	-3	2.5
C14KK	484.7	485.3	1	0.2
C14DK	471.6	472.3	-1	0.5
C16GD	428.5	428.6	-2	0.04
C16GK	441.5	442.3	0	0.04
C16DD	486.5	491.2	-3	0.7
C16KK	512.7	513.3	1	0.08
C16DK	499.6	500.3	-1	0.03
FC14	379.5	-	0	0.12^3^
DDM	510.6	-	0	0.17^3^

1 Theoretical molecular weight (Mw) of lipopeptide detergents.

2 The Mw of lipopetides as determined by mass spectrometry (MS).

3 Values obtained from Anatrace measurements.

### PS-I solubility in the presence of lipopeptides

**Figure 2:**
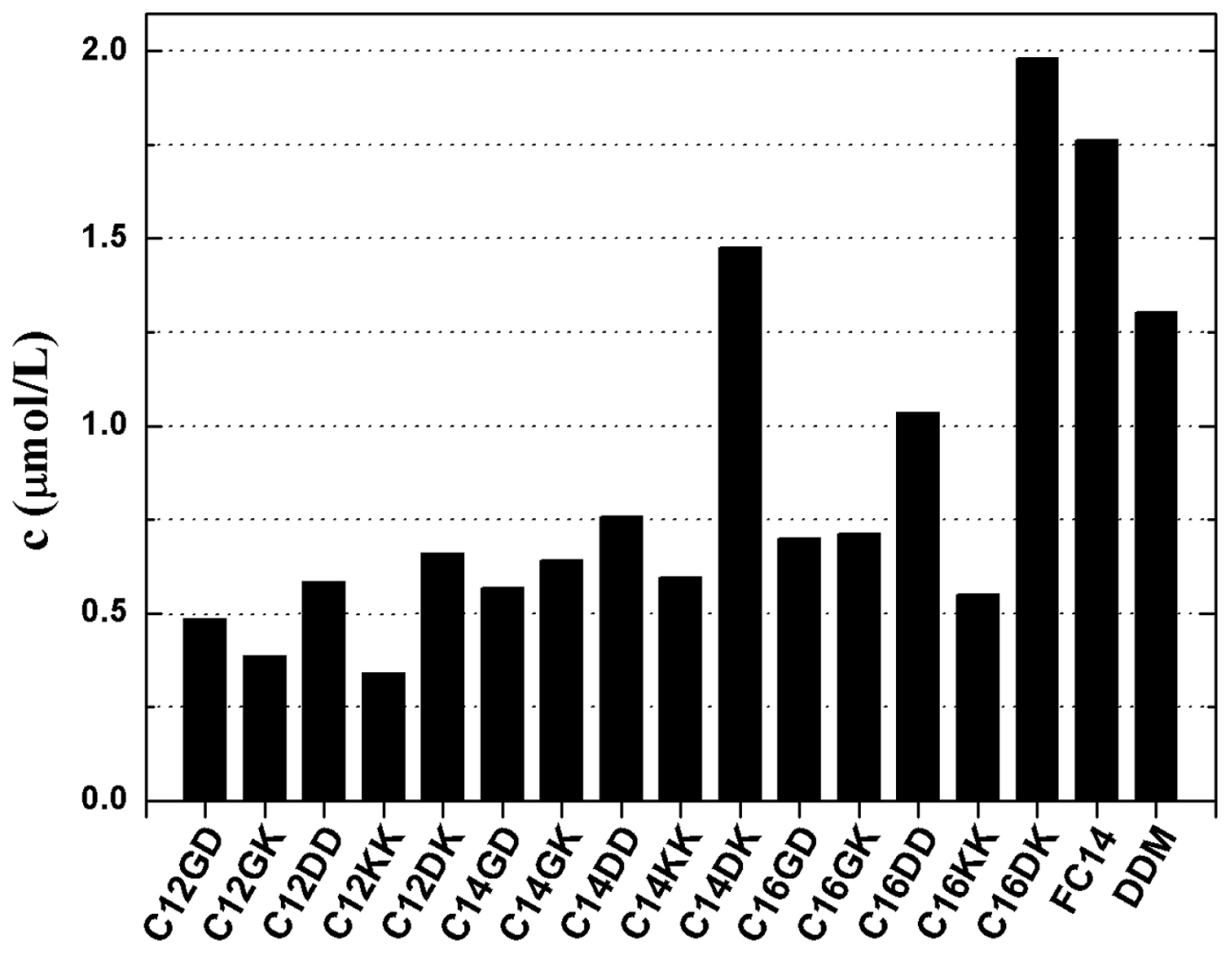
PS-I solubility at the ambient temperature. The values were determined by optical absorption spectroscopy, in the presence of different lipopeptides and conventional surfactants DDM and FC14. All surfactant concentrations were fixed at 5.0 mM. The absorption maximum at 680 nm was utilized to calculate PS-I solubility in surfactant solution, with a molar extinction coefficient of 16.4µM^-1^·cm^-1^. Each bar represents the average of 2 experiments.

PS-I solubility in the presence of lipopeptides was assessed through optical absorption spectroscopy at the room temperature (RT) of 20-23 °C. As an example, the spectrum from 550 to 750 nm of the isolated PS-I solubilized in C14DK is presented in Figure S1, showing a maximum at 680 nm, characteristic of trimeric PS-I. Hence, solubilized PS-I in the final solution was quantified from the Beer-Lambert law based on this peak with a molar extinction coefficient of 16.4µM^-1^·cm^-1^ [26]. Figure 2 shows the PS-I solubility measured in different lipopeptides with the fixed concentration of 5 mM, with the direct comparison of measurements against the 2 conventional surfactants DDM and FC14. As already explained, DDM is a sugar surfactant and FC14 denotes a zwitterionic surfactant with a tetradecyl chain connected to the phosphorylcholine head. Both DDM and FC14 have been shown to be highly effective for solubilizing many membrane proteins and simultaneously maintaining their structural and functional stability [6-8,11]. In contrast, this work has revealed the impressive performance of C14DK and C16DK, whilst most lipopeptides showed much lower efficiencies at solubilizing PS-I than the two conventional surfactants. Among all surfactants tested (including DDM and FC14), C16DK was found to be the most efficient one and was able to solubilize PS-I to a protein concentration of *ca.* 2.0 µmol/L. Note that the solubility of PS-I in the absence of any surfactant was too low to be detectable by absorption spectroscopy.

In general, the ability of lipopeptides to solubilize PS-I was enhanced as the length of their hydrophobic alkyl tails increased from C12 to C16. One exception was C16KK that was less effective than C14KK, yet this might be attributed to the disadvantageous solubility of C16KK in Tris-HCl buffer, which readily precipitated after a prolonged period of time. For the lipopeptide series with the same hydrophobic tails, the one carrying DK showed clear advantage over others in enhancing PS-I solubility.

### Thermostability evaluation of PS-I solubilzed with lipopeptides

**Figure 3:**
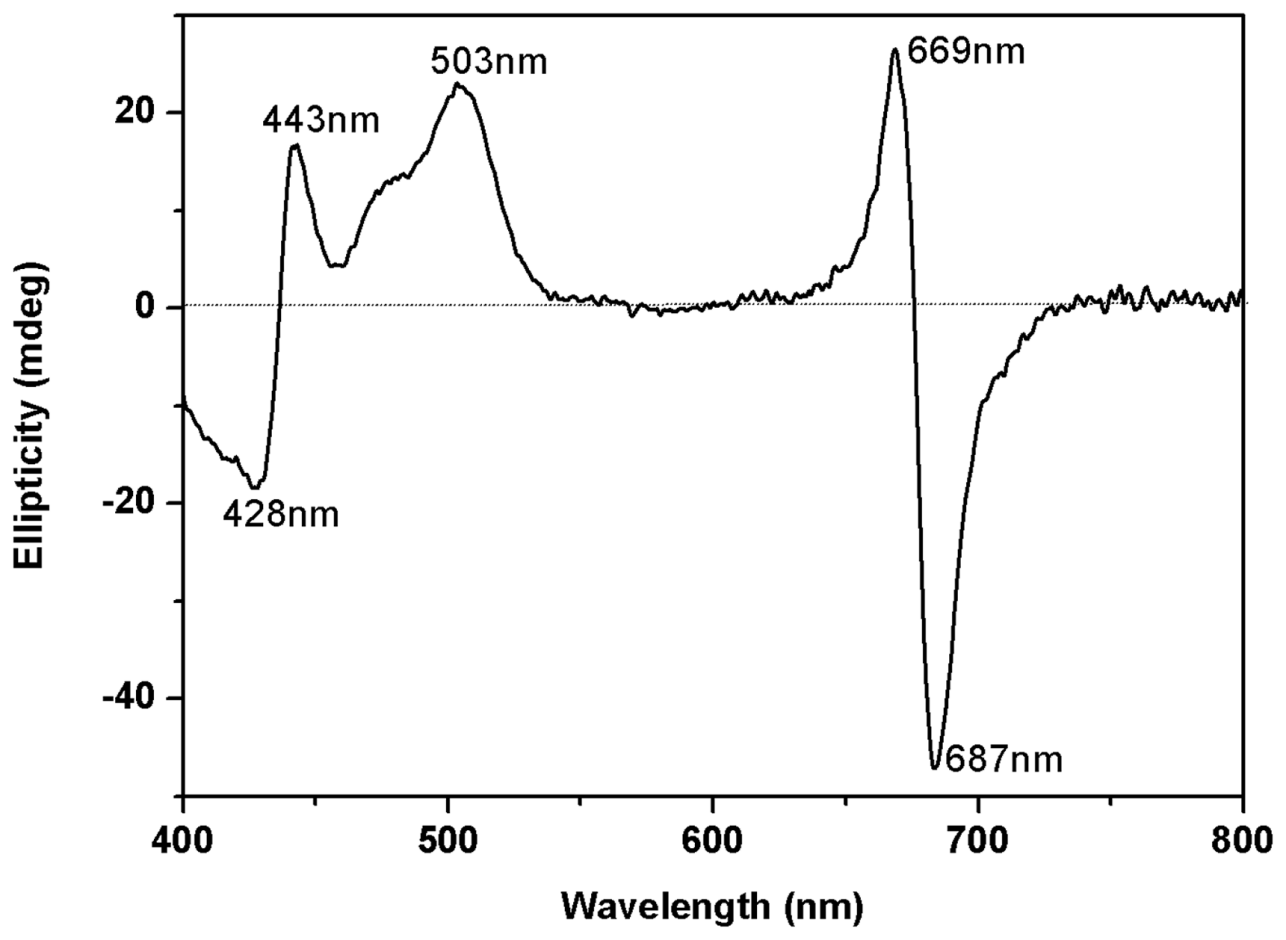
CD spectrum from 400 to 800 nm of PS-I solubilized with 5mM lipopeptide C14DK. The concentration of PS-I is 0.233µmol/L (equal to 20µg Chl/ml).

Thermostability of PS-I solubilized with lipopeptides was assessed through CD spectroscopy. As a typical example, Figure 3 shows the CD spectrum from 400 to 800 nm of PS-I solubilized with C14DK with major bands occurring around 428 (-), 443 (+), 503 (+), 669 (+) and 687 (-) nm. The intensities of these bands vary, with the strongest one around 687 (-) nm. Similar CD spectra were obtained in the presence of other lipopeptides. These results are in good agreement with those previously reported in the CD measurements of PS-I complex [27,28]. As thermal denaturation of PS-I could cause a dramatic decrease in CD intensity at around 687 nm [14], we thus employed the CD signal decrease at this position to evaluate the thermostability of PS-I with different lipopeptides, with the conventional surfactants DDM and FC14 used as control.

Fersht et al have developed a formula to fit the thermal denaturation data as measured from different surfactant solutions [29]. Melting temperatures (T_m_) of PS-I corresponding to the midpoints of the different thermal denaturation curves can be determined by fitting with the formula as given below:

y=(aD+bD*x)*exp(0.1202790474*(x*(Htm/Tm+Cp*ln(x/Tm))-Htm-Cp*(x-Tm))/x)/(1+exp(0.1202790474*(x*(Htm/Tm+Cp*ln(x/Tm))-Htm-Cp*(x-Tm))/x)) +(aN+bN*x)/(1+exp(0.1202790474*(x*(Htm/Tm+Cp*ln(x/Tm))-Htm-Cp*(x-Tm))/x))

where aD is the CD intensity of denatured state, bD is the baseline slope of denatured state, aN is the CD intensity of native state, bN is the baseline slope of native state, Tm is the melting temperature, Htm is the change of enthalpy at Tm and Cp is the change of heat capacity. The whole equation defines the change of free energy in folding-unfolding equilibrium as well as the effect of temperature on its change, which as a result reflects the relative concentrations of native protein and denatured one [29].

**Figure 4:**
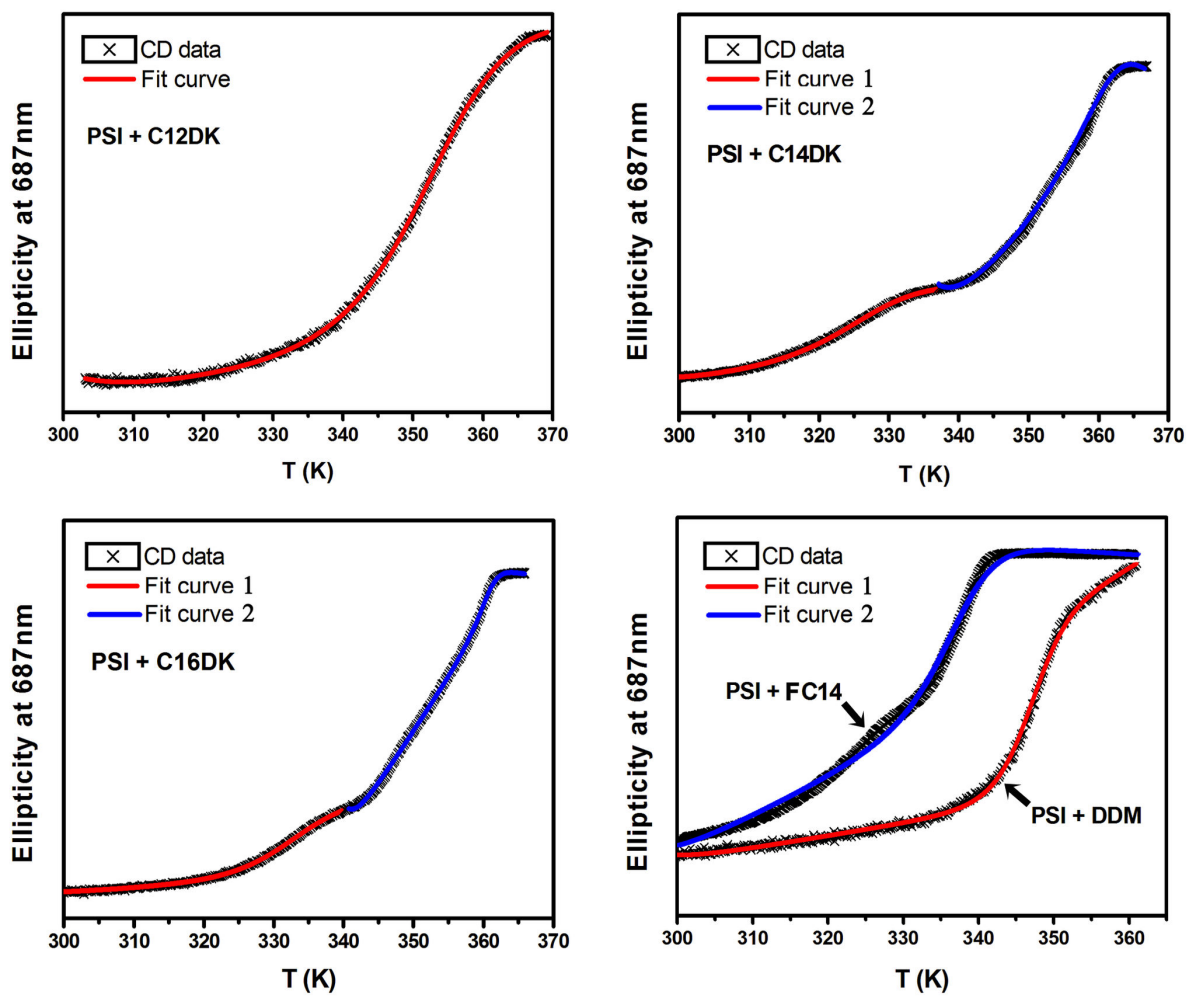
Thermostability of PS-I measured by following ellipticity at 687nm versus temperature. The measurements were conducted in the presence of C12DK, C14DK, C16DK, FC14 and DDM as indicated. The concentrations of PS-I and surfactants were 0.233µmol/L (equal to 20µg Chl/ml) and 5 mM, respectively. The measurement was performed after samples were incubated for 30min at RT. Temperature was increased at a rate of 1K/min. Data points were normalized and fitted with equation 1. An obvious two-stage response was observed in the presence of C14DK and C16DK. Each stage was fitted with equation 1, separately. The best fit curves are as shown in red and blue.

The best fit curves in the presence of C12DK, DDM and FC14 are as shown in Figure 4. In the case of C14DK and C16DK, the two stages were fitted separately. The best fit curves for the first stage are shown in red, while those for the second stage in blue. It is obvious that all the fit curves can well delineate the thermal denaturation changes because of their almost overlap with data points.

Through fitting the denaturation changes, we obtained T_m_ values of PS-I in different detergents that were used to evaluate the thermostability of PSI. Specifically, the T_m_ value of PS-I in lipopeptide C12DK was 352K, which is 5K higher than the T_m_ value of 347K obtained in DDM and 15K higher than the Tm value of 337K obtained in FC14, being indicative of an enhancing effect of thermostability. The two T_m_ values of PS-I obtained through fitting the two-stage response in C14DK were 324K and 361K, and the two T_m_ values obtained in C16DK were 330K and 361K, respectively, showing clear stability enhancements.

### RT fluorescence measurements of PS-I with lipopeptides

**Figure 5:**
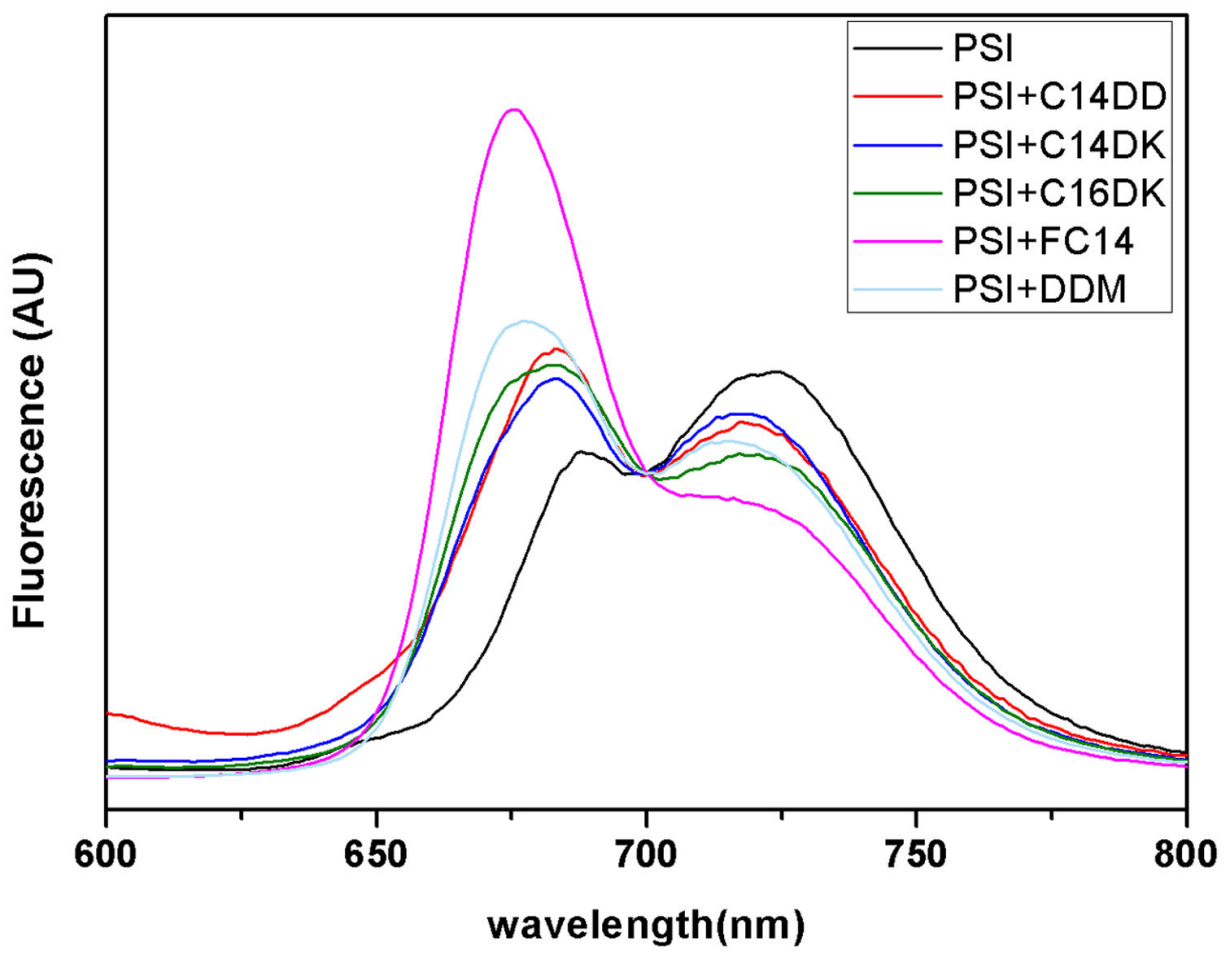
Fluorescence spectra of PS-I at RT in the absence and presence of lipopeptides. All surfactant concentrations were fixed at 2CMC and the concentration of PS-I added was at 0.117µmol/L (equal to 10µg Chl/ml). The spectra are normalized against the peaks 700 nm.

The RT fluorescence emission spectra of PS-I, measured in the absence and presence of lipopeptides, are presented in Figure 5. In the absence of the peptides, PS-I showed a main emission peak at 720 nm and a shoulder peak at 688 nm. Note that although PS-I showed little solubility in Tris-HCl buffer without surfactant, it was able to produce the two fluorescence emission when PS-I samples were vortexed prior to the measurement. The presence of surfactants clearly enhanced the short wavelength peaks. It has been demonstrated that this spectral change resulted from surfactant-induced uncoupling of chlorophyll *a* from the PS-I complex [30]. If this is the case, lipopeptides such as C14DD, C14DK and C16DK imposed an adverse effect on the PS-I structure, although the extent was not as notable as the conventional surfactants such as DDM and FC14 as shown in Figure 5. Hence, lipopeptides may be more favorable for stabilizing the structure of PS-I. Furthermore, the presence of surfactants led to the blue shifting of the short wavelength band with different degrees, with a trend consistent with the band intensity increase. For example, addition of FC14 led to the largest blue shifting to 675 nm, presumably also due to its disadvantageous effect on PS-I structure.

### 77K fluorescence measurements of PS-I with lipopeptide detergents

**Figure 6:**
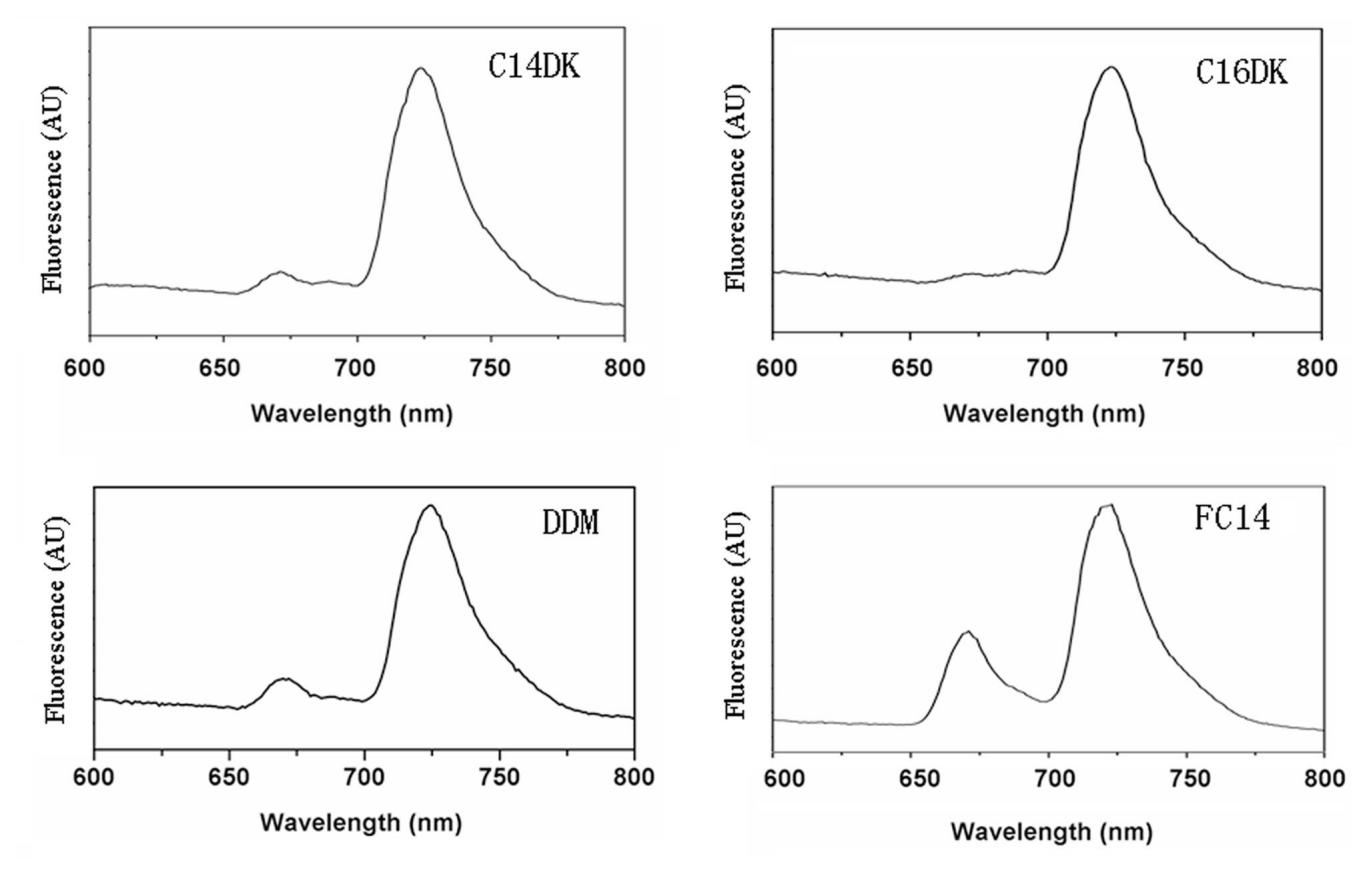
77K fluorescence emission spectra of PS-I solubilized with different surfactants. The surfactants tested include lipopeptides C14DK and C16DK, DDM and FC14 as indicated and the concentrations of surfactants were kept the same as under RT. The concentration of PS-I added was 0.117 µmol/L (equal to 10 µg Chl/ml).

To further assess the effects of different detergents on PS-I structural stability, 77K fluorescence spectra were also recorded. As shown in Figure 6, the emission spectra, obtained through the excitation of chlorophyll (436 nm), were dominated by a peak at 725nm. The spectrum in C14DK also showed an additional peak at about 670 nm though very weak relative to the 725 emission peak. In comparison, the 670 nm emission peak increased clearly in DDM and much more so in FC14 despite the dominance of 725 emission peaks in both surfactants. Similar results have been reported previously, with the appearance of the emission peak in this region attributed to the uncoupled chlorophyll [31]. It is evident that the presence of lipopeptides C14DK and C16DK effectively restrained the 670nm emission peaks. Similar emission spectra were obtained in the presence of lipopeptides C12DK, C14DD and C16DD (data not shown). Hence, lipopeptides are more favorable for the structural stability and integrity of PS-I in comparison with other surfactants.

### Evaluation of the polypeptide composition of PS-I with lipopeptide detergents

**Figure 7:**
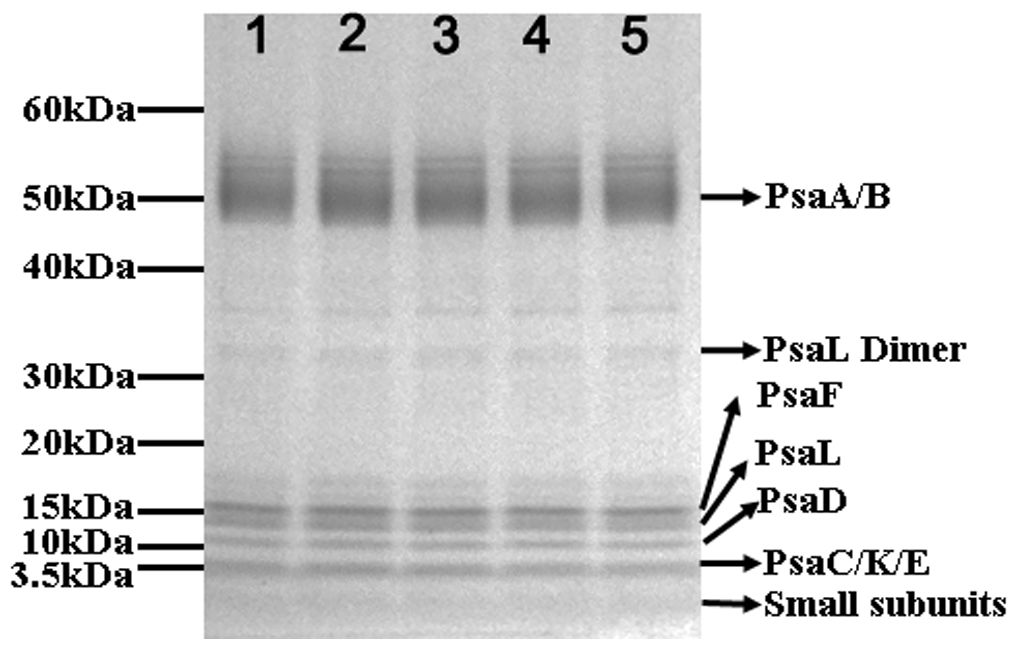
SDS-PAGE analysis of PS-I with different lipopeptides and conventional surfactants. Lane 1, PS-I+C14DD; Lane 2, PS-I+C14DK; Lane 3, PS-I+C16DK; Lane 4, PS-I+DDM; Lane 5, PS-I+FC14.

Apart from the detergent-induced uncoupling of chlorophyll as indicated by fluorescence spectroscopy, the effects of different detergents on the polypeptide composition of PS-I were also analyzed by sodium dodecyl sulfate polyacrylamide gel electrophoresis (SDS-PAGE). As shown in Figure 7, PS-I samples solubilized with lipopeptides C14DD, C14DK and C16DK and conventional detergents DDM and FC14 have similar and complete polypeptide composition. For all PS-I samples, the reaction center subunits PsaA and PsaB are evident at the top of the gel, while other major subunits such as PsaF and PsaL appear routinely as diffuse and weak staining bands. Some small subunits are also vaguely visible below the 3.5kDa band [32]. Hence no other adverse effect of lipopeptides C14DD, C14DK and C16DK on PS-I structure was detectable by SDS-PAGE analysis, since there are full polypeptide bands of PS-I in these lipopeptides in comparison with conventional detergent DDM or FC14.

### Oxygen uptake measurements of PS-I with lipopeptide detergents

**Figure 8:**
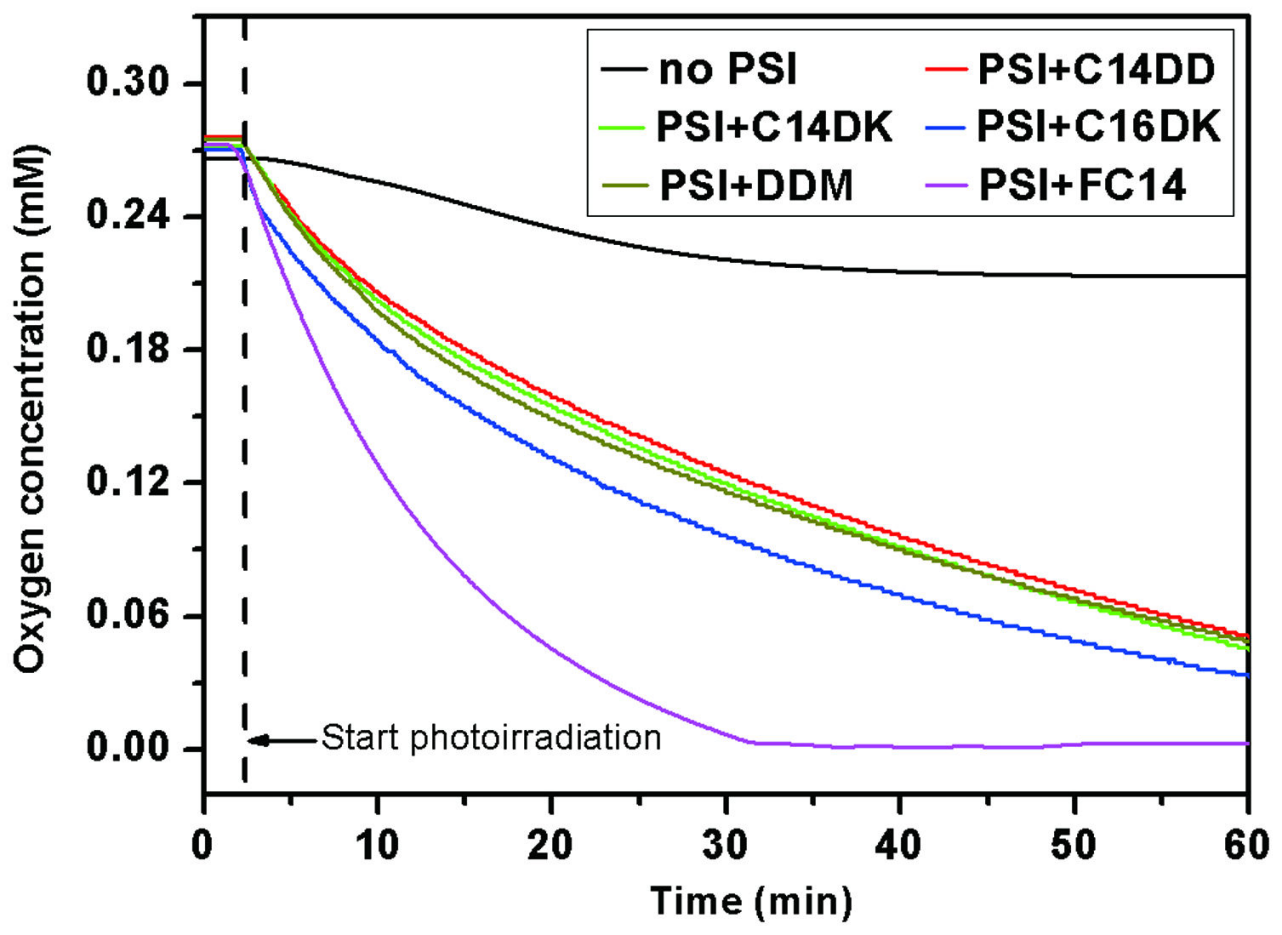
Oxygen uptake activity of PS-I solubilized with different surfactants. The surfactants tested include lipopeptides C14DD, C14DK and C16DK, DDM and FC14 as indicated. The concentration of PS-I added was 0.117 µmol/L (equal to 10 µg Chl/ml). As a control, oxygen uptake of working solution without PS-I was also tested.

Next we studied the effects of lipopeptide detergents on PS-I functionality. The O_2_ uptake activity of PS-I solubilized with different lipopeptide detergents (including C14DD, C14DK and C16DK), DDM and FC14 were measured using a well-established assay [33]. Although a background decrease in dissolved oxygen seems apparent even without PS-I, a clear increase in oxygen uptake upon photoirradiation was observed in all PS-I samples (Figure 8). Based on the initial slope of the decrease in the O_2_ concentration, PS-I samples solubilized with different lipopeptide detergents or with conventional detergents DDM and FC14 showed different biological activities, presumably due to varied effects of detergents on PS-I structure. In spite of the slight differences, the obtained oxygen uptake results of PS-I solubilized with C14DD, C14DK and C16DK indicated successful maintenance of PS-I functionality, since the initial decrease rates in the O_2_ concentration in the presence of these lipopeptides are comparable to that in the presence of DDM. Moreover, it is observed that the decrease rate of O_2_ concentration is much higher in the presence of FC14 than that in the presence of lipopeptide detergents and DDM. It is possible that the presence of FC14 has a synergistic effect on the increase of apparent biological activity of PS-I complex and further investigation will need to be dedicated to gain more insight. However, it should also be mentioned that the presence of FC14 shows considerable adverse effects on the thermostability and structural integrity of PS-I in comparison with lipopeptides and DDM tested, as indicated by CD and fluorescence measurements, and the uncoupled chlorophyll molecules themselves have strong oxygen uptake activity (see Figure S2). Besides, the O_2_ uptake activities of PS-I obtained in the presence of lipopeptide detergents are of the same order of magnitude with those previously reported [12]. These lipopeptide detergents therefore should be promising candidates as new solubilizer for PS-I.

## Discussion

Because of the combined characteristics of lipids and peptides, lipopeptides are promising in many biological applications. Here, a series of simple lipopeptides were designed and synthesized, and subsequently employed to solubilize and stabilize the PS-I membrane protein complex.

To reduce structural complexity and the production cost, dipeptide sequences served as the hydrophilic heads of the designed lipopeptides and through the pair-wise combination of hydrophilic amino acids Gly (G), Asp (D) and Lys (K), five different hydrophilic heads GD, GK, DK, DD and KK were used. The hydrophobic alkyl tails were changed between C12, C14, and C16. The structural variations of these designed lipopeptides notably affected their physiochemical properties including detergency in solution, as reflected by their varied CMCs. As the alkyl chains varied from C12 to C14 and then C16, the CMC decreased almost by a factor of 10 within a given lipopeptide series with the same hydrophilic head. In contrast, variations in the hydrophilic head had less effect on CMC except the ones carrying DD. However, this structural variation was observed to have a significant impact on their solubilizing efficiencies.

Within the designed lipopeptides, C14DK and C16DK showed better solubilizing efficiencies. In comparison to conventional surfactants FC14 and DDM, the solubilizing ability of C14DK was intermediate, although it has a slightly higher CMC of 0.5 mM than the latter two (0.12 and 0.18 mM for FC14 and DDM, respectively). C16DK showed the largest solubilizing capacity among the tested surfactants including FC14 and DDM. Despite having very lower CMC values similar to C16DK, other lipopeptides with the C16 tail (i.e. C16GD, C16GK, and C16KK) displayed markedly lower solubilizing efficiencies than those of C16DK, DDM and FC14. The same trend was also observed for the series with the C14 tail. Hence, the higher solubilizing ability of C14DK and C16DK should not be simply ascribed to their lower CMCs and instead, the composition of the head should be well considered.

During the process of solubilizing membrane proteins, the head groups are located at the interface between surfactant-protein complexes and aqueous solution and also directly interact with the soluble domains of membrane proteins [5]. Thus, they must contribute significantly to protein solubility. Compared with the heads GD and GK, DD, KK, and DK have relative larger sizes. Although DD, KK, and DK carry three charges at circum-neutral pH, their net charges and charge distributions are very different. DD has a net charge of -3 and a charge distribution of --- from N to C terminal, two of which are from the side chain carboxyl groups and one from the C-terminal carboxyl group. KK has a net charge of +1 and a charge distribution of ++-. In contrast, DK has a net charge of -1 and a charge distribution of -+-. The relative large size and even charge distribution of the hydrophilic head DK seem to be able to provide a compatible interface for PS-I in aqueous environment. However, such a hypothesis needs more experimental evidence to support.

In general, surfactants with small ionic heads are harsh and tend to denature membrane proteins, while those with large nonionic or zwitterionic heads are usually mild and non-denaturing [5,9-11]. In spite of being ionic, C14DK and C16DK were observed to more favor the thermal and structural stabilities of PS-I than nonionic DDM and zwitterionic FC14. In the presence of C14DK and C16DK, the two-stage responses of PS-I to temperature increase can be viewed as the composite responses from water-exposed and surfactants-shielded domains. Similar results have been reported previously from denaturant- or ligand-induced protein denaturation [34,35]. The different responses reflect different structural resistances to structural unfolding under denaturing conditions. The sharp transition in C12DK and DDM during thermal denaturation reflected a relatively loose packing between surfactants and hydrophobic domain and thus an overall lower melting of PS-I.

On the whole, our results demonstrated that lipopeptides as designed can enhance the thermostability of the isolated PS-I compared to the conventional detergents like DDM and FC14. Moreover, lipopeptides have obvious advantages in improving PS-I thermostability with the T_m_ for the PS-I systems being shifted up to 326K [14]. As pointed out before, the determination of T_m_ values under protein denaturation is based on the assumption that the system is in reversible equilibrium. Since the denaturation of PS-I is irreversible, the T_m_ values reported here were only nominal and should not be interpreted as a parameter for thermodynamic stability. However, they are still useful for checking the influence of lipopeptides on the thermostability of PS-I [14].

The structural stability of PS-I solubilized in lipopeptides was also investigated by fluorescence spectroscopy. Lipopeptides posed less negative effects on the structure of multi-subunit PS-I complex in comparison with DDM and FC14, based on the changes of fluorescence emission spectra that were assigned to surfactants-induced uncoupling of chlorophylls [30,31]. However, such changes could also be associated with the existence of the solubilized PS-I in different forms, e.g., monomeric or trimeric. The aggregating states might also vary under different surfactants [36]. Nevertheless, our results in the present work suggest that these designed lipopeptides are mild; whilst they improve solubility they do not cause notable adverse structural changes of the PS-I, evident from the fluorescence studies and SDS-PAGE analysis. Moreover, O_2_ uptake measurements suggest these lipopeptides do not compromise PS-I functionality. Hence these designed lipopeptides are favorable for the structural stability and integrity of the multi-subunit PS-I complex and even other membrane proteins.

## Materials and Methods

### Synthesis of Lipopeptide Detergents

Lipopeptide detergents used in this work are comprised of a hydrophilic head and a hydrophobic tail. The molecular structures of the designed lipopeptides are shown in Figure 1, which are characterized by the coupling of the alkyl chains of lauric acid, myristic acid and palmitic acid (termed C12, C14 and C16, respectively) as hydrophobic tails onto the N termini of the peptide backbones by amide bonding. The synthesis of these lipopeptides was described in our previous work [37]. In brief, the lipopeptides were synthesized from the Wang resin by using the standard Fmoc solid-phase synthesis strategy on a CEM, Liberty microwave peptide synthesizer. Lauric acid, myristic acid or palmitic acid was introduced separately following the sequential coupling of amino acids onto the Wang resin from C terminus to N terminus. The carboxylic groups were activated by treatment with HBTU/HOBt/DIEA. Upon completion of lipopeptide synthesis, cleavage from the resin and deprotection of the side chains of amino acids were performed concurrently with a mixture of triflouroacetic acid, triisopropylsilane and H_2_O at a ratio of 95:2.5:2.5. The cleavage mixture was rotary evaporated to a concentrated solution. The raw product was subsequently dissolved into chloroform and extracted by mixing with water using a separation funnel. After rotary evaporation of chloroform, the product was collected and then dissolved in methanol and further extracted by adding petroleum ether. After being extracted by petroleum ether several times, the lipopeptides were collected by evaporating the methanol phase and then lyophilized for 2 days. The lyophilized lipopeptides were subjected to reverse-phase HPLC and analyzed by mass spectrometry. The purity of the lipopeptides obtained was higher than 95%.

### Determination of CMCs of Lipopeptides

To determine the critical micelle concentrations (CMC) of the lipopeptides, surface tension method was employed. For each lipopeptide, 30 serial dilutions were prepared in 20mM Tris-HCl buffer (pH 7.5). The surface tension measurements were carried out with the EasyDyne tensiometer (Kruss) at 25.0±0.1°C by using the Wilhelmy plate method. The values of surface tension γ were determined after a period of 30 min. The lipopeptide concentration at which surface tension did not exhibit any appreciable change with further increase of concentration was regarded as the CMC value.

### Isolation of PS-I Complex

The PS-I complex was extracted from the thylakoid membranes of *Spirulina* *platensis* following the procedures described previously [38]. Algal cells were harvested and suspended in STNMC solution (300 mM sucrose, 10 mM NaC1, 5 mM MgCl_2_, 5 mM CaCl_2_, 50 mM Tris-HCl and pH 7.8) with protease inhibitor phenylmethyl sulfonyl fluoride (PMSF, 20 mM). The cells were sonicated and cell debris was removed by centrifugation at 3000 g for 15 min. The photosynthetic membrane protein complex was then isolated by centrifugation at 50000 g for 60 min. The membranes were washed and solubilized in buffer (50 mM Tris-HCl pH 7.8, 5 mM MgCl_2_, 5 mM CaCl_2_) containing nonionic surfactant Triton X-100 at a concentration that was 22.5 times that of Chla (chlorophyll-a). The supernatant was loaded onto a 10–30% (w/v) step sucrose gradient at 4°C and centrifuged for 16 h at 160000 g. The lower green band was collected and stored at -20°C. The purified PS-I was characterized with SDS-PAGE, fluorescence spectrum and circular dichroism (CD). The chlorophyll content of PS-I was measured as described in [39,40].

### Determination of PS-I Solubility in Surfactants

Isolated PS-I samples were treated with solutions containing 5 mM surfactant in 20 mM Tris-HCl buffer (pH 7.5) at RT for 12 hours. The solubilized PS-I samples were separated from the insoluble part by centrifugation at 13000 rpm for 1.5 min in a Sigma mini-centrifuge. The supernatants thus obtained were measured by optical absorption spectroscopy from 550 nm to 750 nm. The absorption maximum at 680 nm was employed to calculate PS-I solubility in surfactant solution according to the Beer-Lambert law, with a molar extinction coefficient of 16 µM^-1^·cm^-1^ [26]. All experiments were carried out in duplicate.

### Thermostability Evaluation of PS-I Solubilzed with Surfactants

The absorption of red form chlorophylls is strongly dichroic and the conformational changes can be detected with visible CD signal between 600-760 nm [41]. The decrease of the CD signal in this region indicated the dimerization of red chlorophylls and a decrease in the efficiency of energy transfer from antenna to reaction center [42,43]. This CD signal is more specific and sensitive than that at 190–250 nm. The thermal stability of the PS-I complex solubilized was studied on a Bio-Logic MOS-450 CD spectrometer. CD spectra from 400 nm to 800 nm were first recorded at RT for the PS-I samples solubilized with different lipopeptides with a final concentration of 0.233 µmol/L (equal to 20 µg Chl/ml). Samples were incubated for 30 min prior to recording the CD spectra using 10 mm path length fused quartz cuvettes. The spectra were recorded in 1 nm steps with an integration time of 0.2 s and a band-pass of 2 nm. Melting temperatures of PS-I in the presence of different lipopeptides or DDM were determined by measuring the CD signal at 687 nm as a function of temperature [14]. The temperature was automatically controlled between 25°C and 95°C through a Peltier device with an increase rate of 1 °C/min. The concentrations of surfactants used in all cases were at 5 mM.

### Room Temperature (RT) Fluorescence Emission Spectra

RT fluorescence spectra of PS-I samples solubilized with different surfactants were measured on a spectrofluorimeter (F-2500, Hitachi, Tokyo, Japan) with 436 nm as the excitation wavelength. The fluorescence emission spectra were recorded from 600 to 800 nm with slit width set to 5 nm. The pathlength of the cell was 5 mm. Before each measurement, each PS-I sample with surfactant was centrifuged at 13000 rpm for 1.5 min in a Sigma mini-centrifuge to remove insoluble particles. The detergents were used at the concentrations of 2CMC. The concentration of PS-I added was at 0.117 µmol/L (equal to 10 µg Chl/ml).

### 77K Fluorescence Emission Spectra

77K fluorescence spectra of PS-I samples solubilized with different surfactants were also measured on a spectrofluorimeter (F-2500, Hitachi, Tokyo, Japan) using a liquid nitrogen cryostat. The fluorescence emission spectra were recorded from 600 to 800 nm, with 436 nm as the excitation wavelength, and the slit width set to 2 nm. The pathlength of the cell was 6 mm. The detergents were used at 2CMC. The concentration of PS-I added was 0.117 µmol/L (equal to 10 µg Chl/ml).

### SDS-PAGE analysis

The structural integrity of PS-I solubilized with different detergents was analyzed by SDS-PAGE. To remove any unbound subunits, PS-I samples were prepared by 5 concentration-dilution cycles with solubilization buffer (20mM Tris-HCl, pH 7.5 plus detergent) through a Millipore 100, 000Da MW cutoff filter. PS-I samples were then loaded in Novex 4-12% Bis-Tris SDS-PAGE gel (Invitrogen) according to standard protocols except the samples were incubated at room temperature prior to loading because boiling caused membrane protein aggregation. After resolved on SDS-PAGE gel (run in NuPAGE MOPS buffer at 100V), protein samples were visualized using silver staining (a more sensitive alternative to Coomassie; Invitrogen)

### Oxygen uptake measurements

Measurements of the O_2_ uptake activity of PS-I were conducted at 20 °C using a Clark-type O_2_ electrode (Hansatech Instruments, England). White light from a 100 W halogen lamp through a 1/4 neutral density filter was used to illuminate a 1-cm diameter reaction vessel of volume 2.5 mL. A buffer containing 20 mM Tris-HCl (pH 7.5) supplemented with 0.2 mM dichloroindophenol (DCIP) and 5 mM sodium ascorbate as an electron donor couple and 0.2 mM MV^2+^ as an electron acceptor was used for O_2_ uptake measurements. An aliquot of a sample solution was added to the buffer just before measurements. The concentration of PS-I solubilized with 2CMC surfactant was 0.117 µmol/L (equal to 10 µg Chl/ml). The O_2_ uptake activity was estimated from the initial slope of the decrease in the O_2_ concentration upon illumination.

## Supporting Information

Figure S1 **Optical absorption spectrum of isolated PS-I solubilized in C14DK, with a Chl concentration of 10 µg/ml.** The absorption maximum at 680nm was indicated.
(TIF)

Figure S2 **Oxygen uptake measurement of chlorophyll *a* (1µg/ml) solubilized with DDM.**
(TIF)
