# Supplementary figures and images for: Solubilization and Stabilization of Isolated Photosystem I Complex with Lipopeptide Detergents
Source: PLoS One. 2013 Sep 30;8(9):e76256. doi: 10.1371/journal.pone.0076256 (PMC3787008; doi:10.1371/journal.pone.0076256)

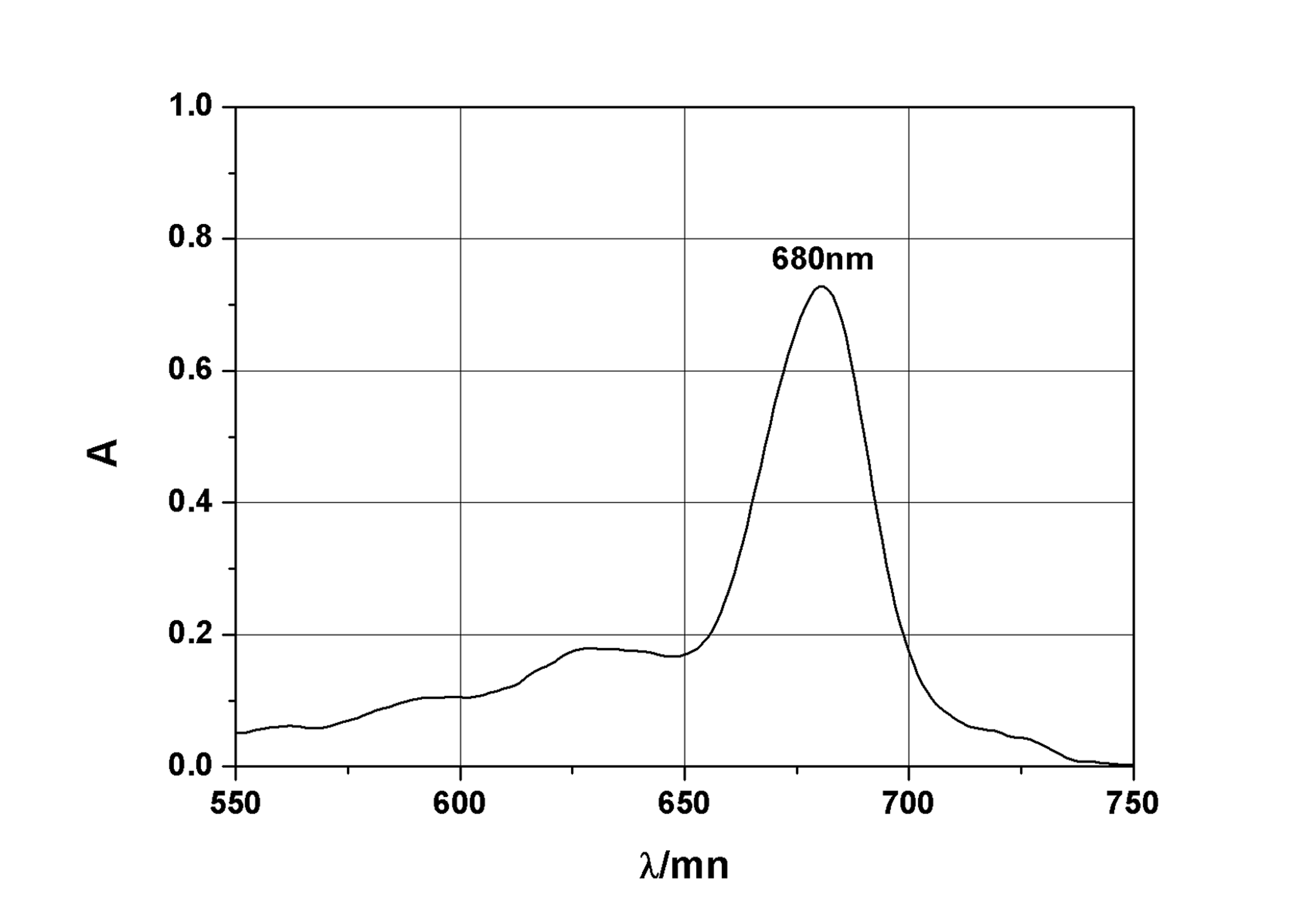

Supplement: Figure S1 — Optical absorption spectrum of isolated PS-I solubilized in C14DK, with a Chl concentration of 10 µg/ml. The absorption maximum at 680nm was indicated. (TIF) [file pone.0076256.s001.tif]
